# Simulation-Based Evaluation of Treatment Adjustment to Exercise in Type 1 Diabetes

**DOI:** 10.3389/fendo.2021.723812

**Published:** 2021-08-19

**Authors:** Julia Deichmann, Sara Bachmann, Marie-Anne Burckhardt, Gabor Szinnai, Hans-Michael Kaltenbach

**Affiliations:** ^1^Department of Biosystems Science and Engineering and Swiss Institute of Bioinformatics (SIB), ETH Zurich, Basel, Switzerland; ^2^Life Science Zurich Graduate School, Zurich, Switzerland; ^3^Pediatric Endocrinology and Diabetology, University Children’s Hospital Basel, and Department of Clinical Research, University Hospital Basel, University of Basel, Basel, Switzerland

**Keywords:** type 1 diabetes, modeling, physical activity, exercise, treatment adjustment, insulin sensitivity, hypoglycemia

## Abstract

Regular exercise is beneficial and recommended for people with type 1 diabetes, but increased glucose demand and changes in insulin sensitivity require treatment adjustments to prevent exercise-induced hypoglycemia. Several different adjustment strategies based on insulin bolus reductions and additional carbohydrate intake have been proposed, but large inter- and intraindividual variability and studies using different exercise duration, intensity, and timing impede a direct comparison of their effects. In this study, we use a mathematical model of the glucoregulatory system and implement published guidelines and strategies *in-silico* to provide a direct comparison on a single ‘typical’ person on a standard day with three meals. We augment this day by a broad range of exercise scenarios combining different intensity and duration of the exercise session, and different timing with respect to adjacent meals. We compare the resulting blood glucose trajectories and use summary measures to evaluate the time-in-range and risk scores for hypo- and hyperglycemic events for each simulation scenario, and to determine factors that impede prevention of hypoglycemia events. Our simulations suggest that the considered strategies and guidelines successfully minimize the risk for acute hypoglycemia. At the same time, all adjustments substantially increase the risk of late-onset hypoglycemia compared to no adjustment in many cases. We also find that timing between exercise and meals and additional carbohydrate intake during exercise can lead to non-intuitive behavior due to superposition of meal- and exercise-related glucose dynamics. Increased insulin sensitivity appears as a major driver of non-acute hypoglycemic events. Overall, our results indicate that further treatment adjustment might be required both immediately following exercise and up to several hours later, but that the intricate interplay between different dynamics makes it difficult to provide generic recommendations. However, our simulation scenarios extend substantially beyond the original scope of each model component and proper model validation is warranted before applying our *in-silico* results in a clinical setting.

## Introduction

Type 1 diabetes (T1D) is a common endocrine disorder that results from autoimmune destruction of pancreatic β-cells and leads to elevated blood glucose levels (hyperglycemia) if untreated. Treatment consists of exogenous insulin administration to cover dietary carbohydrate intake and needs to be tailored to each individual with continuous adjustments over time. A basal insulin level is provided either by continuous subcutaneous infusion using an insulin pump or once or twice daily injection of long-acting insulin to maintain glucose homeostasis in fasting conditions. In addition, bolus injections of rapid-acting insulin are used to compensate for meals, where the required dose depends on the size of the meal, the blood glucose level immediately preceding the meal, and potentially the insulin-on-board from previous injections.

Regular physical activity (PA) is beneficial for people with T1D and is therefore recommended in current clinical guidelines (1, 2). However, physical activity leads to dynamic changes in blood glucose regulation on two different time-scales: first, increased energy requirements by working muscles lead to increased glucose uptake and corresponding faster decrease of blood glucose during the activity (3–5). Second, insulin sensitivity increases during the activity and remains elevated for several hours during subsequent recovery to help replenish glycogen stores (6). Clinical guidelines recommend reducing the insulin bolus for a pre-exercise meal as well as additional carbohydrate intake before and during exercise depending on the initial blood glucose level respectively the duration of the exercise (7, 8), as well as a potential reduction in basal insulin. However, accurately adjusting a person’s treatment to physical activity remains a largely unsolved problem in general (9), both immediately following exercise (acute hypoglycemia) and several hours later, particularly overnight (late-onset hypoglycemia). Indeed, fear of exercise-induced hypoglycemia is a major impediment for patients to exercise regularly (10).

Clinical guidelines for adjusting treatment to physical activity are based on evidence from a plethora of clinical trials. Similarly, newer proposals, e.g., to exploit the ability to detect glucose trends using continuous glucose monitoring (CGM) devices are thoroughly evaluated using clinical trials. On the other hand, the large heterogeneity of patient responses and many degrees of freedom to define study protocols—such as pre-exercise meals and specification of the exercise session—might make it difficult to evaluate the differences between adjustment strategies directly. These factors also pose challenges when summarizing results over different studies. In addition, preventing both acute and late-onset hypoglycemia might induce competing goals for an adjustment strategy. For example, recent proposals to use high-intensity intervals before exercise were shown to decrease acute hypoglycemia (11, 12), but can increase the risk of late-onset hypoglycemia (13).

Furthermore, clinical guidelines must strike a balance between accuracy and ease of use by patients, and typically use discrete categories for adjustments depending on intensity and duration of exercise. This can lead to abrupt changes in treatment adjustment for very similar exercise scenarios at the ‘border’ of categories.

On the other hand, mathematical models have been used in diabetes research and care for several decades (14, 15). Suitable models allow *in-silico* clinical trial simulation to improve trial design (16). Model-based *in-silico* evaluation of control strategies in the context of automated insulin delivery systems is also becoming increasingly important and first systems gained FDA approval for design, testing, and validation of closed-loop controllers (17), which allows *in-silico* evaluation and optimization of control methodologies (18, 19) and treatment adjustments (20, 21). Several models also consider the effects of physical activity on glucose dynamics (22–25).

Here, we use a mathematical model to directly compare the effect of several published exercise-related adjustment strategies *in-silico*. We consider a broad variety of scenarios and consider different moderate exercise intensities and durations, but also exercise times in relation to adjacent meals and to bedtime. We compare the strategies’ performances with relevant summaries such as time-in-range and acute and late-onset hypoglycemia risk. This setup thus provides insight into the individual and combined effects of different exercise modalities.

We rely on published components for our model, but did not validate the full model for the considered scenarios. Our conclusions are therefore tentative and an exact quantification of the comparisons depends on the accuracy of the underlying mathematical model. Nevertheless, our simulations are in line with clinical experience, show clear qualitative differences between the guidelines under different exercise scenarios, and point at areas requiring further clinical study. In particular, while all strategies can substantially reduce the risk of acute hypoglycemia, prolonged changes in insulin sensitivity would require additional insulin bolus adjustment for meals following exercise to avoid late-onset hypoglycemia. Moreover, the timing of exercise in relation to meals can lead to inadvertent effects, particularly when considering glucose trends.

## Methods

We rely on published models for our *in-silico* study of treatment adjustments to physical activity and provide a detailed description of the final model in the following section. We consider two published treatment adjustment strategies and compare them using standard performance measures. We also propose exploiting ideas from global sensitivity analysis (GSA) and review the necessary methodology in the final methods section.

### Model

We use a published validated model for glucose-insulin regulation that captures the changes to glucose metabolism driven by short moderate-intensity exercise (22) to simulate the expected blood glucose dynamics with and without treatment adjustments. We augment this model with a published extension to account for the intensity- and duration dependence of exercise-driven changes in insulin sensitivity (23). We allow for glucose appearance after a meal using a published model describing the appearance rate (26). Finally, we describe insulin kinetics after injection of a subcutaneous bolus using a published two-compartment model (27) that we calibrated on published data for insulin as part (28) using ordinary least squares regression.

The glucose-insulin dynamics proposed in (22) are given by the system of ordinary differential equations

(1) $$\begin{array}{l} \dot{X}=-p_{2}\cdot X+p_{3}\cdot \Delta I\\ \begin{array}{l} \dot{G}=-p_{1}\cdot (G-G_{b})-(1+\alpha \cdot W\cdot Z)\cdot X\cdot G-\alpha \cdot W\cdot Z\cdot X_{b}\cdot G\\ \;-\beta \cdot Y\cdot G+\frac{Ra}{V_{g}\cdot BW} \end{array}\\ \dot{Y}=-\frac{1}{\tau_{HR}}\cdot Y+\frac{1}{\tau_{HR}}(HR-HR_{b})\\ \dot{Z}=-\left(f(Y)+\frac{1}{\tau }\right)\cdot Z+f(Y), \end{array}$$

where a dot denotes a time-derivate and we suppress the explicit dependence on time in our notation. The state *X* [1/min] describes the action of insulin in a remote compartment on plasma glucose *G* [mg/dl], *G_b_* is the basal plasma glucose level, and *p_1_* to *p_3_* are rate parameters. Insulin is considered as the difference Δ*I* = *I* – *I_b_* = *I_t_* + *I_c_* – *I_b_* between plasma insulin concentration *I* [µU/ml] and the basal level *I_b_*. Plasma insulin is divided into the concentration *I_t_* required to achieve a target glucose level *G_t_* and a contribution *I_c_* from insulin bolus injections.

The model considers the increase in heart rate *HR* [bpm] above its basal rate *HR_b_* to quantify exercise intensity, and encodes the cumulative effect delayed by a time constant *τ_HR_* [min] in a state variable *Y*, representing energy expenditure. This impacts the glucose concentration through a state *Z* driven by the function

(2) $$f(Y)=\frac{{\left(\frac{Y}{a\cdot HR_{b}}\right)}^{n}}{1+{\left(\frac{Y}{a\cdot HR_{b}}\right)}^{n}},$$

defining the exercise onset when *Y* reaches a certain fraction *a* of the basal heart rate *HR_b_*. The time constant *τ* [min] allows for a slow decay of *Z* after exercise.

We follow a proposal by Dalla Man et al. (23) and account for the dependence of the exercise-driven rise in insulin sensitivity on exercise duration and intensity using the integrated over-basal heart rate

(3) $$W=\int_{0}^{t}\left(HR-HR_{b}\right)\;dt.$$

Overall, exercise increases insulin action by the factor *α*·*W*·*Z*. This includes the increase in the effect *X_b_* of basal insulin on plasma glucose, which was also incorporated by (23). The insulin-independent rise in glucose clearance is given by *β*·*Y*·*G* and is proportional to the exercise intensity.

We use an established model to describe the appearance rate *Ra* [mg/min] of glucose in plasma after carbohydrate intake (26):

(4) $$Ra=\frac{f\cdot D\cdot t\cdot e^{-t/\tau_{max}}}{\tau_{max}^{2}},$$

where *f* is the bioavailability of the meal, *D* [mg] is the amount of ingested carbohydrates (CHO) and *τ_max_* [min] is the time of maximum appearance rate. The parameters *V_g_* [dl/kg] and *BW* [kg] give the glucose distribution volume and the body weight, respectively.

Finally, we use a two-compartment model described in (27) to describe insulin kinetics after a subcutaneous injection:

(5) $$\begin{array}{l} {\dot{x}}_{1}=-k_{21}\cdot x_{1}+u\\ {\dot{x}}_{2}=k_{21}\cdot x_{1}-(k_{d}+k_{a})\cdot x_{2}\\ {\dot{I}}_{c}=\frac{k_{a}}{V_{i}\cdot BW}\cdot x_{2}-k_{e}\cdot I_{c}, \end{array}$$

where the injected insulin u[µU/min] passes through the subcutaneous compartments *x_1_* and *x_2_* [µU] before reaching the plasma insulin compartment; *k_21_, k_d_, k_a_* and *k_e_* are rate parameters and *V_i_* [ml/kg] is the insulin distribution volume.

We provide the parameters of this model in **Table 1**. The parameters of the glucose-insulin model were taken from the original publication (22), but we adjusted α based on the model augmentation proposed in (23). For the parameters of the glucose appearance rate, we used the original parameters given in (26). Finally, we calibrated the insulin injection model to insulin aspart using ordinary least squares regression based on recently published data (28). Throughout this study, we consider an ‘average’ person of *BW* = 70kg body weight with a resting heart rate of *HR_b_* = 80bpm and basal insulin requirements of *I_b_* = 10µU/ml and a target glucose of *G_t_* = 120mg/dl.

**Table 1:** Model parameters.

Parameter	Value	Unit
**Glucose regulation** (22)		
*p_1_*	0.0041	1/min
*p_2_*	0.0155	1/min
*p_3_*	6.913 · 10^-6^	1/min^2^ per *µ*U/ml
*G_b_*	172	mg/dl
*α*	2.59 · 10^-4^	dimensionless
*β*	3.39 · 10^-4^	1/bpm
*τ_HR_*	5	min
*τ*	600	min
*a*	0.1	dimensionless
*n*	4	dimensionless
**Meal** (26)		
*f*	0.8	dimensionless
*V_g_*	1.6	dl/kg
*τ_max_* (slow)	60	min
*τ_max_* (fast)	20	min
**Insulin** (28)		
*k_21_*	0.0085	1/min
*k_d_*	0.0247	1/min
*k_a_*	0.011	1/min
*k_e_*	0.0357	1/min
*V_i_*	104	ml/kg

Parameter α was adjusted according to the model extension in (23).

### Insulin Bolus Calculation

We calculate the insulin bolus u[U] required to compensate a given meal according to

(6) $$u=\frac{CHO}{ICR}+\frac{G-G_{t}}{CF},$$

where *CHO*[g] is the amount of carbohydrates in the meal, *G* [mg/dl] is the current glucose level (in the simulation) and *G_t_* [mg/dl] denotes the target glucose level. We round the calculated insulin dose to the nearest 0.5 U to mimic the usual practice in MDI-therapy. Throughout, we ignore a potential correction for insulin-on-board, since insulin injections are sufficiently spaced over time in all simulations.

In practice, the insulin bolus calculation is adjusted to an individual using the patient-specific insulin-to-carbohydrate ratio, *ICR* [g/U], and a patient-specific correction factor *CF* [mg/dl/U]. For our simulations, we consider a single ‘typical’ person and fix these two parameters to *ICR* = 15g/U and *CF* = 20mg/dl/U, which results in good glycemic control together with the model parameters in **Table 1**.

### Treatment Adjustment Guidelines

For our simulation studies, we consider three treatment adjustment scenarios based on two sets of recommendations. First, we use the decision-tree carbohydrate intake algorithm developed in (29) that uses continuous glucose monitoring to propose ingestion of CHO during the activity based on glucose concentration and trend. Second, we use a collection of recommendations given in recent consensus guidelines (7) that address insulin and CHO requirements before, during and after exercise. In many cases, the guidelines provide a range rather than a specific amount of recommended CHO intake, and we reflect this in our simulations by separately considering the low and high end of the proposed range.

#### Carbohydrate Intake Algorithm

The carbohydrate intake algorithm was proposed by Riddell and Milliken and exploits information from real-time CGM to improve glucose levels during exercise and avoid hypoglycemia (29). Carbohydrate intake is recommended once glucose drops below 126 mg/dl and exercise is suspended if glucose falls below 70 mg/dl.

Specifically, 8 g CHO are recommended for glucose between 110 and 126 mg/dl and dropping at a rate greater than 5.4 mg/dl per 5 min (as indicated on a CGM device). For lower glucose levels between 90 and 110 mg/dl, the algorithm recommends 16 g CHO if glucose drops between 5.4–9.9 mg/dl per 5 min (indicated by one downward arrow) and increases the recommendation to 20 g CHO if it drops faster than 9.9 mg/dl per 5 min (indicated by two downward arrows). Finally, the algorithm proposes ingestion of 16 g CHO independent of the rate of glucose change for glucose levels below 90 mg/dl.

For our simulations, we allow multiple intakes of fast-acting CHO during the activity, but require a minimum time of 20 min between successive snacks.

#### Consensus Guidelines

A recent consensus statement provides a detailed overview on exercise management, including glucose targets, carbohydrate recommendations and insulin dose adjustments for both bolus and basal insulin for different forms of exercise (7). Here, we restrict consideration to the recommendations pertinent to exercise between 30–120 min at moderate intensity.

For moderate-intensity exercise, the guidelines recommend a proportional reduction of the meal insulin bolus (Eq. 6) depending on the exercise intensity and duration if a meal was eaten within 120 min before exercise is started (**Table 2**).

**Table 2:** Consensus guidelines: recommended bolus insulin reduction for pre-exercise meal if bolus is administered within 120 min of exercise onset.

$\%\;VO_{2}^{\max}$	HR [bpm]	Exercise duration	
		30–60 min	>60 *min*
25–50	100–130	–25%	–50%
50–70	130–155	–50%	–75%
≥70	≥155	–75%	–75%

Additional glucose targets and carbohydrate intake strategies are given to stabilize BG levels at the onset of exercise: exercise is only started if BG is above 90 mg/dl, and CHO intake is recommended based on the BG level and the insulin condition, such that a higher CHO intake is required if insulin concentrations are high.

The guidelines distinguish between low and high insulin conditions to modify the recommended CHO intake during exercise, but do not define these conditions in detail. We consider a simulation in the high insulin condition if insulin is injected at most 120 min prior to exercise onset and in the low insulin condition otherwise. For our simulations, we separately consider following the low respectively high end of the recommended range of carbohydrate intake as given in **Table 3**.

**Table 3:** Consensus guidelines: CHO intake before, during and after exercise.

	Insulin Condition	
	low	high
**At exercise onset based on BG**		
<90 mg/dl	15 g	25 g
90–124 mg/dl	10 g	10 g
>124 mg/dl	—	—
**During exercise based on duration**		
≤30 min	—	15–30 g
30–60 min	10–15 g/h	30–60 g/h
> 60 min	30–60 g/h	up to 75 g/h
**At end of exercise based on BG**		
<90 mg/dl	20 g	20 g
≥90 mg/dl	—	—

If BG is <90 mg/dl at exercise onset, exercise is delayed until BG>90 mg/dl. During exercise of more than 60 min under high insulin conditions, we consider CHO intake of 60–70 g/h.

The guidelines also generically recommend a meal after exercise. For our simulations, we follow clinical experience and consider the intake of 20 g CHO without insulin bolus if BG is below 90 mg/dl immediately following exercise.

### Performance Measures

In addition to direct comparisons of predicted blood glucose curves, we consider time-in-range (TIR) and the low (LBGI) respectively high (HBGI) blood glucose index as three well-established measures to quantify the performance of different treatments.

The time-in-range gives the percentage of time that an individual’s glucose concentration remains in the desired range of 70–180 mg/dl. The TIR does not distinguish between low and high blood glucose excursions.

The low blood glucose index (LBGI) and the high blood glucose index (HBGI) measure the extent and frequency of low, respective high blood glucose events based on the BG risk function (30), given as

(7) $$r(G)=10\cdot 1.509^{2}\cdot {\left[{\left(\text{I}n\;(G)\right)}^{1.084}-5.381\right]}^{2}.$$

This function provides a quantitative risk score for each BG level. It has a minimum at 112.5 mg/dl, and it is customary to distinguish values below and above this minimum *via*

(8) $$\begin{array}{llll} rl(G) & = & \{\begin{array}{l} r(G)\\ 0 \end{array} & \begin{array}{l} \text{if}\;G<112.5\;\text{mg}/\text{dl}\\ \text{otherwise} \end{array}\\ rh(G) & = & \{\begin{array}{l} r(G)\\ 0 \end{array} & \begin{array}{l} \text{if}\;G\geq 112.5\;\text{mg}/\text{dl}\\ \text{otherwise}. \end{array} \end{array}$$

Given *n* blood glucose readings *G*_1_, …, *G_n_*, the LBGI and HBGI correspond to the average risk of the recorded events below respectively above the threshold of 112.5 mg/dl:

(9) $$\begin{array}{lll} \text{LBGI} & = & \frac{1}{n}\sum_{i=1}^{n}rl(G_{i})\\ \text{HBGI} & = & \frac{1}{n}\sum_{i=1}^{n}rh(G_{i}) \end{array}$$

### Variance-Based Global Sensitivity Analysis

We use a global sensitivity analysis (GSA) approach (31) to investigate how simultaneously changing model parameters and the timing of exercise is reflected in changes in the time-in-range and low blood glucose index. Our rationale is that parameters in the mathematical model are associated with physiological processes; large variation caused by parameters associated with the same process would then allow us to gauge the relative importance of different processes on the TIR and LBGI under exercise.

Global sensitivity analysis is a standard tool for evaluating identifiability and robustness of nonlinear models (32). For a given function *f*(*p*_1_, *p*_2_, … ,*p_k_*) with *k* inputs, GSA varies all inputs simultaneously and records the resulting responses. We can then calculate the variance *V* of the responses. The first-order Sobol index *S*_1_(*i*) of the *i*th input is the proportion of *V* attributed to variation in *p_i_* alone (also called the main effect), while the second-order Sobol index *S*_2_(*i*) is the proportion additionally attributed to co-variation (or second-order interaction) of *p_i_* and any other parameter. The total Sobol index *S_T_*(*i*) describes the overall contribution of parameter *p_i_* to the variation in the response, considering its main effect and all interactions of any order.

Intuitively, a large *S_T_*(*i*) results if variation of input *p_i_* yields large variation in the response; the indices *S*_1_(*i*) and *S*_2_(*i*) then further detail what part of this variation is caused by *p_i_* alone respectively in conjunction with simultaneous variation of another input.

## Results

We base our treatment adjustment comparisons on a simulated standard day using the model outlined in above. Our standard day consists of a 24h simulation period, starting at 6:00h in the morning. We consider three typical meals: a breakfast of 50 g carbohydrates at 7:00h, a lunch of 70 g carbohydrates at 13:00h, and a dinner of 60 g carbohydrates at 19:00h. For each meal, we set the time of maximum appearance rate to *τ_max_* = 60min; this corresponds to a typical mixed meal. We administer an insulin bolus injection 15 min before each meal and use the bolus calculator (Eq.6) to determine the required standard bolus. For simulations based on the consensus guidelines, we decrease this bolus according to **Table 2** if required. We modify our standard day for some of our simulation scenarios and indicate these changes in the corresponding sections.

### Variation of Insulin Sensitivity and Meal Appearance

**Figure 1:**
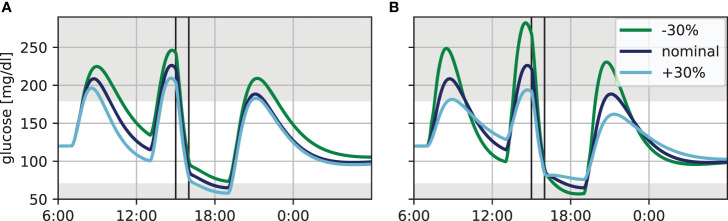
Variation in blood glucose dynamics for the standard day with 60 min of exercise at a heart rate of 120 bpm (vertical lines). **(A)** Insulin sensitivity varied by ±30%. **(B)** Time of maximum glucose appearance *τ_max_* varied by ±30%.

It is well known that insulin sensitivity varies strongly between individuals. We therefore asked how the glucose dynamics changes with changes to the basal insulin sensitivity in our simulation in the presence of exercise. For this, we simulated blood glucose trajectories for our standard day, but added a 60 min exercise session at a very moderate heart rate of 120 bpm at 15:00h. We then altered the basal insulin sensitivity by ± 30% without adjusting the treatment and keeping patient-specific parameters *ICR* and *CF* constant, resulting in the dynamics shown in **Figure 1A**. The impact of insulin sensitivity is clearly visible and within the range expected from clinical experience. Noteworthy, increasing the insulin sensitivity (i.e. increasing the parameter *p_3_*) shifts the glucose levels before and during exercise, but yields virtually identical BG levels after exercise (**Figure 1A** dark blue and light blue).

Next, we considered the impact of glucose appearance from meals using the same exercise scenario. Specifically, the time *τ_max_* to maximum appearance is highly variable in practice and strongly depends on the meal composition, which is often not known precisely. We therefore altered this parameter by ±30%, corresponding roughly to meals with moderately slow respectively moderately fast glucose appearance (**Figure 1B**). Altering glucose appearance has a strong effect on the height and width of the resulting blood glucose peak following the meal. Slower appearance, corresponding to a more complex meal composition and described by a higher *τ_max_*, leads to low and broad peaks, while appearance of fast-acting CHO is characterized by high and narrow peaks. Furthermore, the decrease in BG levels after the peak is more pronounced for smaller *τ_max_*, since the meal is already absorbed while the bolus insulin is still active.

Differences in insulin sensitivity and in meal absorption therefore complicate a direct comparison of treatment options based on blood glucose trajectories of different individuals and meals. In the following, we therefore fix the insulin sensitivity and use a fixed time of maximum glucose appearance of 60 min for the main meals in order to isolate the treatment effects and allow a direct comparison of different treatment adjustments to exercise.

### Timing of Physical Activity

The effect of exercise on blood glucose depends on exercise duration and intensity but also on the timing between exercise and meals, which affects resulting BG dynamics. However, the impact of exercise performed during different phases of meal absorption on BG is not obvious and guidelines only consider insulin bolus reductions for pre-exercise meals. To further elucidate the relation between meal absorption and exercise, we examined the effect of exercise timing on blood glucose levels with and without applying treatment adjustments.

We again base our simulations on the standard day described in the beginning of this section. In addition, exercise was performed for 60 min at a heart rate of 120 bpm. We considered several starting times for the exercise session, starting at 13:30h (immediately following lunch) and spaced in 30 min steps until a latest session at 17:30h that finishes 30 min before dinner.

**Figure 2:**
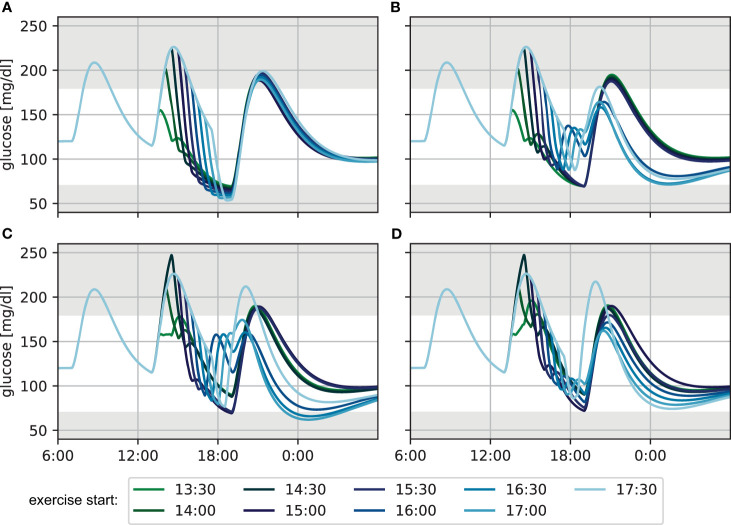
Glucose levels for exercise performed at different times during the afternoon for 60 min at a heart rate of 120 bpm **(A)** without treatment adjustment to exercise, **(B)** with adjustment based on the CHO algorithm, **(C)** using the lower CHO recommendation of the consensus guidelines and **(D)** using the upper CHO recommendation of the consensus guidelines.

We compare the resulting blood glucose trajectories without adjustments, with adjustment following the carbohydrate intake algorithm, and with adjustment following the consensus guidelines with low respectively high carbohydrate intake if required. We assumed a fast glucose appearance with parameter *τ_max_* = 20min for the suggested carbohydrate snacks. The resulting trajectories are shown in **Figure 2**.

Without treatment adjustment to exercise (**Figure 2A**), exercise sessions later after lunch start at comparatively lower BG levels, as more of the meal carbohydrates are already absorbed at the time of exercise. Consequently, later exercise leads to lower minimal BG levels towards the end of the session, reaching hypoglycemia in many cases. At the same time, the overall decrease in BG level is considerably higher for exercise started more closely to lunch, because the exercise-independent decrease of blood glucose from meal absorption and the active insulin from the meal bolus combine with the increased glucose demand during exercise. While BG levels are higher at the end of exercise for these earlier exercise sessions, the meal glucose is continued to be absorbed after the activity and glucose keeps decreasing. Consequently, blood glucose trajectories are similar for all exercise scenarios by dinner time. Moreover, exercise-driven insulin sensitivity returns to baseline only slowly over the course of several hours during recovery, and the difference in exercise timing has little impact on the overnight BG curves. Nevertheless, the increased insulin sensitivity manifests in a considerably lower blood glucose of about 100 mg/dl during the night, as compared to the unperturbed target level of 120 mg/dl at the beginning of the simulation.

When applying the CHO intake algorithm (**Figure 2B**), the BG trajectories fall into two broad categories. For earlier exercise, the algorithm proposes only a small amount of CHO towards the end of the session, and BG further decreases to 70 mg/dl between exercise and dinner. For later exercise, on the other hand, BG levels fall below 90 mg/dl during the activity and the algorithm correspondingly recommends a larger CHO amount which results in considerably higher BG concentrations at dinner time compared to the previous case. This difference in BG at dinner time leads to substantial changes in the calculated meal insulin bolus: adjustments for later exercise result in a higher calculated insulin bolus because they stabilize the BG level during and after exercise and yield BG levels close to the target at dinner time. This bolus amplifies the elevated effect of insulin on glucose disappearance and results in low overnight BG. In contrast, adjustments for earlier exercise also stabilize BG levels during exercise, but yield low BG levels at dinner time. The calculated insulin bolus is now reduced, resulting in an overnight BG in the normoglycemic range.

Next, we adjusted treatment following the consensus guidelines and used the low recommended CHO amount during exercise (**Figure 2C**). For early exercise sessions, the guidelines result in a reduced insulin bolus for lunch. Then, BG levels can be maintained during the activity and stay in the normoglycemic range until dinner. When exercise is performed in the afternoon starting at 15:00 or 15:30, the insulin bolus is not reduced, BG drops strongly during exercise and reaches 70 mg/dl by dinner time. For later exercise, the guidelines recommend additional intake of 20 g CHO without insulin bolus and BG consequently increases after the activity and remains high when the dinner bolus is administered. Similar to the CHO intake algorithm, the differences in BG level at dinner time and associated differences in insulin bolus maintain normoglycemia overnight for early- and mid-afternoon exercise, but result in low BG for the other scenarios.

Finally, we considered the upper end of the recommended CHO amount for the consensus guidelines (**Figure 2D**). The additional carbohydrates push the blood glucose higher during exercise compared to the previous scenario and result in more similar BG levels at dinner time for the different timings. However, the insulin bolus calculation still yields slightly different recommended doses, and while overnight BG trajectories now all remain in the normoglycemic range, higher insulin doses for later sessions result in gradually lower BG trajectories.

**Figure 3:**
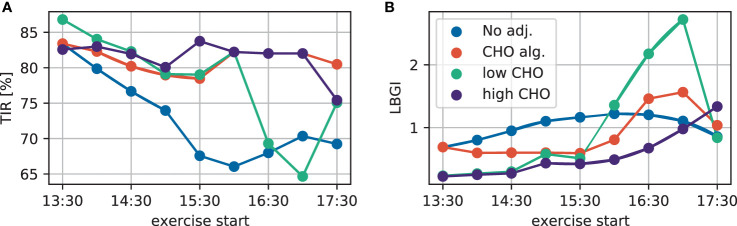
**(A)** TIR and **(B)** LBGI for different exercise starting times considering no adjustment (blue), the CHO intake algorithm (orange), and the consensus guidelines with low (green) and high (purple) CHO intake.

To summarize the performance of the treatment adjustments and allow more direct comparison, we evaluate the time-in-range (TIR) and low blood glucose index (LBGI) over a 24-hour period resulting from each scenario (**Figure 3**). Without treatment adjustment, TIR is lower when exercise is started later, but increases again slightly for exercise starts after 16:00h. All three treatment adjustment strategies lead to substantial improvement of TIR and show similar results for the first two hours. Most notably, the consensus guidelines with low CHO intake show substantial deterioration when starting exercise after 16:00h, moving closer towards dinner time, in agreement with the trajectories in **Figure 2**.

Comparing the treatment adjustments in terms of LBGI yields a similar overall conclusion: without adjustment, LBGI continuously increases the later exercise is started. While the consensus guidelines show very similar results both for the lower and higher CHO recommendation for early exercise, using the low end of the proposed CHO intake results in higher LBGI for later exercise.

Overall, the consensus guidelines with high CHO intake give the best performance both in terms of TIR and LBGI for this simulation scenario. The qualitatively similar results for TIR and LBGI over all adjustments indicate that adjustments mainly improve TIR by avoiding hypoglycemia, without overly increasing hyperglycemia in the process.

### Combination of Exercise Intensity, Duration and Timing

The two sets of treatment adjustment strategies explicitly consider duration and intensity of the exercise, but do not account for the time of exercise during the day or with respect to adjacent meals, unless exercise is performed within two hours of a meal. Given the substantial impact of this timing on the blood glucose trajectories, we next look at combinations of exercise duration, intensity, and timing, and evaluate the resulting BG trajectories with and without treatment adjustments. We now consider exercise at three moderate intensities of *HR* = 120, 140 and 160bpm and vary the exercise duration in 15 min increases from 30 min up to 120 min. Finally, we consider each combination of intensity and duration in three scenarios based on our standard day, where we alter the starting time of the exercise session to enforce a bolus insulin reduction, respectively a low and high insulin condition. We again use *τ_max_* = 20 min to model the fast absorption of exercise snacks.

**Figure 4:**
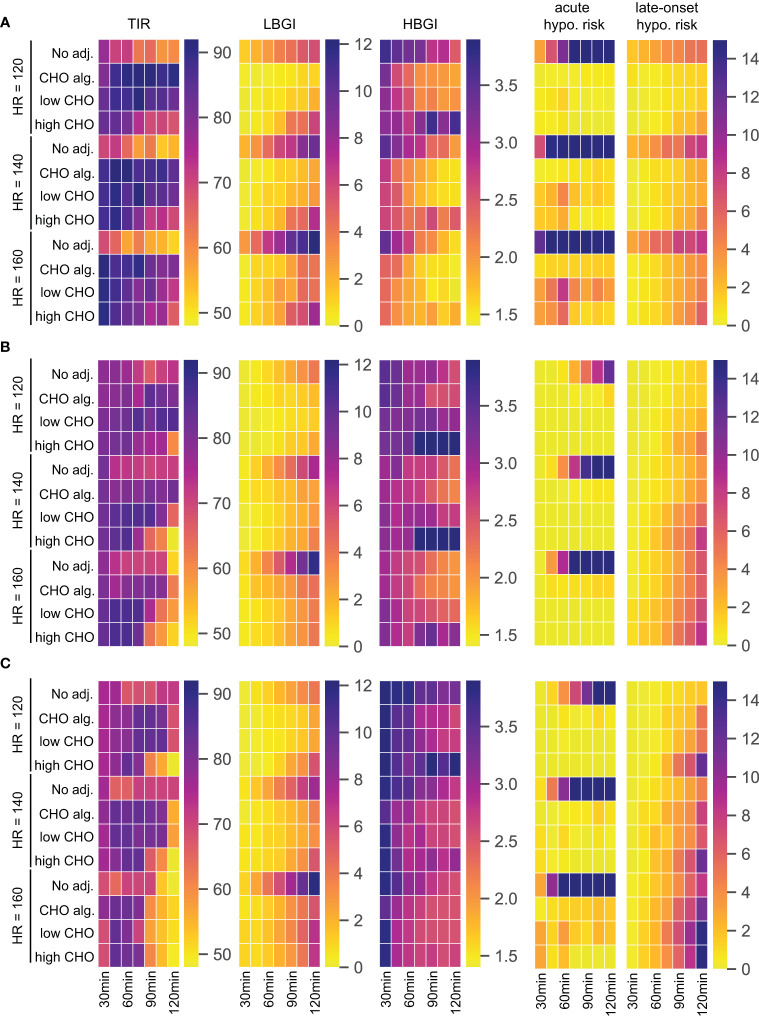
TIR, LBGI and HBGI over 24h-simulations and the corresponding acute and late-onset hypoglycemia risk for different adjustments, intensities, and exercise durations. **(A)** Scenario 1: exercise in postabsorptive state. **(B)** Scenario 2: exercise with prior insulin bolus reduction. **(C)** Scenario 3: exercise without prior insulin bolus reduction.

We again consider no adjustment to treatment, the carbohydrate intake algorithm, and the consensus guidelines using the low respectively high end of the recommended CHO intake. We summarize the performance of each treatment adjustment using time-in-range, and low and high blood glucose index. Since a main focus of treatment adjustment is avoidance of exercise-induced hypoglycemia, we additionally quantify the risk of acute and late-onset hypoglycemic episodes for each scenario and calculate the LBGI of acute hypoglycemia during and up to 60 min after exercise, and the LBGI of late-onset hypoglycemia during the night from 19:00h to 6:00h the following morning. We summarize the simulation results as heatmaps shown in **Figure 4**. Note that for TIR, larger values with darker more red-pink colors indicate better time-in-range, while for the other four measures lower values with lighter more yellow colors indicate better treatment.

#### Scenario 1: Exercise in Postabsorptive State

For our first scenario, we modify our standard day and introduce an exercise session starting at 12:00h while postponing lunch to 14:30h. Then, the BG level is close to fasting levels at the beginning of exercise and glucose dynamics are entirely governed by exercise and corresponding treatment adjustments. The performance measures are shown in **Figure 4A**.

For this scenario, all three treatment adjustments show substantial improvements over no adjustment for TIR and hypoglycemia-related LBGI, acute and late-onset hypoglycemia risk measures. Time-in-range is high using any adjustment, showing only a slight decrease towards longer exercise duration for high CHO intake. For both TIR and LBGI, the carbohydrate intake algorithm and the consensus guidelines with low CHO intake show comparable results. Interestingly, using the consensus guidelines with high CHO intake results in higher LBGI for longer exercise duration compared to the other two adjustments. This adjustment also shows inferior HBGI control, indicating that the high CHO intake likely overcompensates for the glucose uptake from exercise, resulting in higher HBGI, while being unable to maintain a sufficient blood glucose level over longer periods.

All three strategies achieve near-prevention of acute hypoglycemia for lower intensity exercise regardless of duration, and the carbohydrate intake algorithm manages to keep acute hypoglycemia risk low also for higher intensities. Meanwhile, both variants of the consensus guidelines show increased acute risk for higher intensities, especially for durations around one hour.

Risk of late-onset hypoglycemia is substantial in this scenario for higher intensities and longer duration. All three adjustment strategies reduce this risk and we observe a clear dose-response relation with longer duration and higher intensity being more difficult to control, with the carbohydrate intake algorithm and the consensus guidelines with low CHO intake reducing the risk slightly more than following the high CHO recommendations.

#### Scenario 2: Exercise With Prior Insulin Bolus Reduction

Next, we modify our standard day by introducing an exercise session at 14:30h, corresponding to a time shortly after lunch. The consensus guidelines then propose a reduction of the insulin bolus for lunch, and exercise is performed in a high insulin condition. The results are given in **Figure 4B**.

Again, all three treatment adjustments improve LBGI and acute hypoglycemia risk compared to no adjustment over all durations and intensities. While the CHO intake algorithm shows good overall control of TIR, applying the consensus guidelines results in a decrease of TIR for longer and more intense exercise, especially for high CHO intake. This is explained by the corresponding increase in HBGI for these scenarios, which exceeds a no treatment option substantially and indicates a likely overcompensation with too high CHO amounts.

The carbohydrate intake algorithm shows slightly elevated acute hypoglycemia risk compared to the two consensus guidelines variants, but simultaneously presents a lower risk for late-onset hypoglycemia. Notably, late-onset hypoglycemia risk is similar for no adjustment and the CHO algorithm, while it increases compared to no adjustment for the consensus guidelines and longer exercise duration. In addition, long exercise of higher intensity poses a problem for all three strategies: while acute hypoglycemia risk is well-controlled, the risk of late-onset hypoglycemia increases rapidly with intensity and duration.

#### Scenario 3: Exercise Without Prior Insulin Bolus Reduction

As our third scenario, we modify our standard day by an exercise session at 15:30h. Blood glucose is then still elevated from the preceding lunch at the beginning of exercise. However, consensus guidelines do not adjust the insulin bolus for lunch due to the larger time gap between meal and exercise, and exercise is performed in a low insulin condition. We show results in **Figure 4C**.

In this scenario, all three treatment adjustments struggle to keep time-in-range high for higher intensities and longer durations, and consensus guidelines with high CHO intake show low TIR for longer exercise even at low intensity, with corresponding high HBGI for these exercise scenarios. In contrast to the previous scenarios, HBGI is generally high, and particularly so for short duration exercise.

Reduction of LBGI compared to no adjustment is clearly visible for all strategies, but works less efficient compared to scenario 2. As before, LBGI deteriorates for all adjustments with increasing intensity and duration.

The risk of acute hypoglycemia in this scenario is very high without treatment adjustment, even for comparatively short duration of exercise. All three adjustment strategies reduce this risk to very low values, with notable increase in risk for the highest intensity. The carbohydrate intake algorithm shows good control of acute hypoglycemia risk throughout, and consistently yields the lowest risk of late-onset hypoglycemia. Meanwhile, the two consensus guideline adjustments help reduce the acute risk further, and the high CHO intake in particular is very successful for higher intensities and longer duration in this regard. On the other hand, better reduction of acute hypoglycemia results in increased risk of late-onset hypoglycemia. Overall, late-onset hypoglycemia seems difficult to control in this scenario for all adjustments, and the risk score is substantially higher compared to the two previous scenarios, and reaches very high levels for long exercise duration.

In summary, all three adjustment strategies show dramatic improvement in all measures compared to no adjustment. The consensus guidelines are successful in avoiding acute hypoglycemia when exercise is preceded by a meal, but less so in a postabsorptive state. The carbohydrate intake algorithm also performs well in this regard. While all three adjustment strategies also reduce the risk of late-onset hypoglycemia when starting exercise in a postabsorptive state, all strategies struggle to maintain a low late-onset risk score when blood glucose dynamics from a preceding meal are added. Of note in these scenarios is that all strategies show higher late-onset risk score compared to no adjustment, clearly indicating the difficulties to maintain glycemic control over a long period with multiple factors impacting the blood glucose dynamics. Indeed, preventing acute and late-onset hypoglycemia appear to be conflicting goals, with better acute control leading to worsened late-onset control.

### Application of Guidelines to a Patient Population

**Figure 5:**
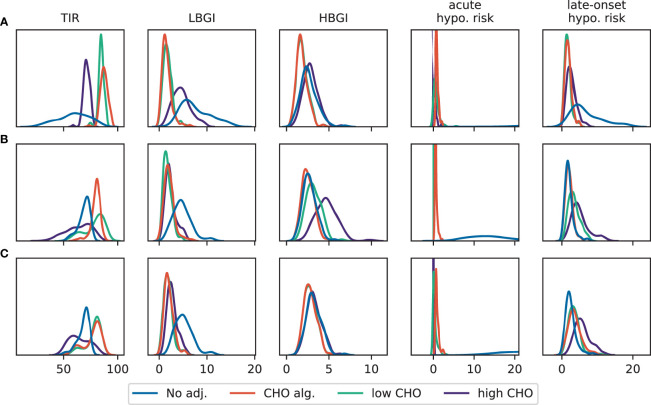
Distribution of TIR, LBGI and HBGI and the corresponding acute and late-onset hypoglycemia risk for different adjustments from 24h-simulations of a patient population. Exercise is performed for 90 min with *HR* = 140bpm. **(A)** Scenario 1: exercise in postabsorptive state. **(B)** Scenario 2: exercise with prior insulin bolus reduction. **(C)** Scenario 3: exercise without prior insulin bolus reduction.

To evaluate whether our conclusions generalize to a broader population, we repeat our analysis for 100 new subjects, described by different parameter sets. We allow variation in the most important parameters glucose effectiveness (*p_1_*), insulin sensitivity (*p_3_*), exercise-driven increase in insulin sensitivity (α) and glucose clearance (β), and sample each parameter independently from a corresponding normal distribution with a standard deviation of 20% of the nominal parameter value. We again consider the three scenarios described previously and concentrate on an exercise duration of 90 minutes and an intensity of *HR* = 140bpm for brevity. The results are shown in **Figure 5**, where we observe excellent agreement with our previous conclusions for all five performance measures and all adjustments. We also considered the remaining exercise durations and again found excellent agreement with our conclusions from the ‘typical’ individual (**Supplementary Figures S1–S3**), including outcomes on the individual subject level (shown for 10 randomly selected subjects in **Supplementary Figures S4–S6**).

### Sensitivity Analysis

Our final analysis aims at quantifying which physiological processes drive the time-in-range and LBGI in the presence of exercise. For this, we exploit ideas from global sensitivity analysis to decompose the observed variation in TIR, respectively LBGI, into components associated to individual model parameters. We again use our standard day and add a 60 min exercise session at 15:30h, with a very moderate intensity of *HR* = 120bpm.

We used the Python package SALib (33) for calculating the Sobol indices. We allowed each parameter to vary up to ±20% around its nominal value (**Table 1**), and uniformly sampled *N* = 51,000 random parameter sets from the resulting parameter region. For each sampled parameter set, we simulated the blood glucose trajectories without further treatment adjustments and recorded TIR and LBGI, before calculating the variation of these two responses using the standard variance estimator.

**Figure 6:**
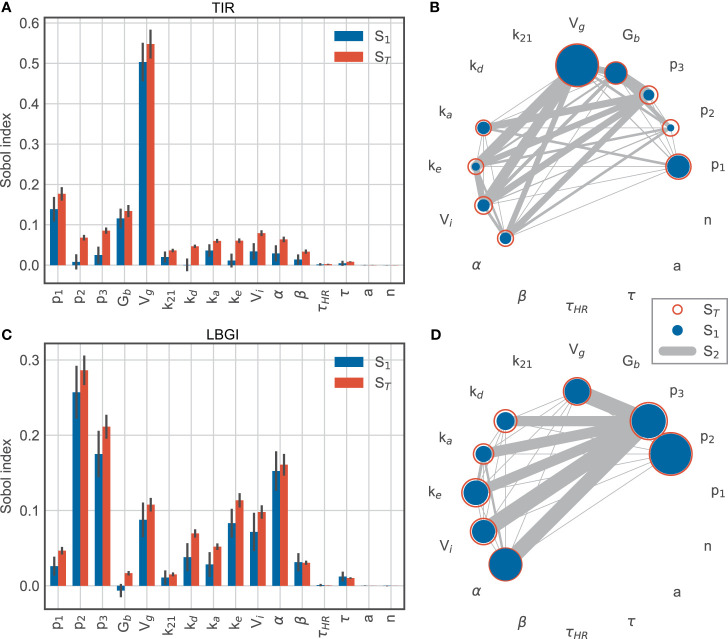
Sobol sensitivity indices for time-in-range (top row) and low blood glucose index (bottom row). **(A, C)** main effect *S_1_* and total effect *S_T_*. **(B, D)** second-order effects *S_2_* for parameters with a minimum total effect of 5%.

For time-in-range, we find that the glucose distribution volume *V_g_* has the largest impact (**Figure 6A**), with a first-order sensitivity of about 50%. In other words, changes in the distribution volume account for about half of the variation in the observed TIR for this simulation scenario. This is not surprising, as *V_g_* largely determines the BG rise after meal intake and hence affects hyperglycemic episodes. The glucose effectiveness *p_1_* and the basal glucose concentration *G_b_* provide the second- and third-largest contribution with main effects of about 10% each. Thus, glucose-related parameters explain the vast majority of variation in TIR. In addition, we find substantial interactions between the insulin action parameters *p_2_* and *p_3_* and the insulin kinetics parameters *k_a_*, *k_e_* and *V_i_* (**Figure 6B**), as well as between *p_2_* and *p_3_* with α, which scales the exercise-driven increase in insulin sensitivity. While these parameters only account for a smaller fraction of the observed variation in TIR individually, the substantial interactions between these parameters indicate an intricate interplay of insulin-related processes.

In contrast, the variation in low blood glucose index is mainly associated with insulin-related parameters, while the importance of the glucose distribution volume *V_g_* is much lower than for TIR (**Figures 6C, D**). Specifically, *p_2_* and *p_3_* dominate the explained variation with first-order effects of 26% and 18%, respectively. These parameters are associated with insulin-driven glucose disappearance from plasma and thus affect hypoglycemic BG excursions. In addition, exercise-driven changes in insulin sensitivity captured by α contribute to this effect during and after exercise, with a large main effect of 15%. Compared to TIR, the LBGI is more affected by insulin kinetics represented by *k_d_*, *k_e_* and *V_i_*.

Together, these results suggest that TIR is affected mainly by glucose-related processes, likely due to hyperglycemic excursions following meals, while parameters related to insulin action explain most of the variation in LBGI.

**Figure 7:**
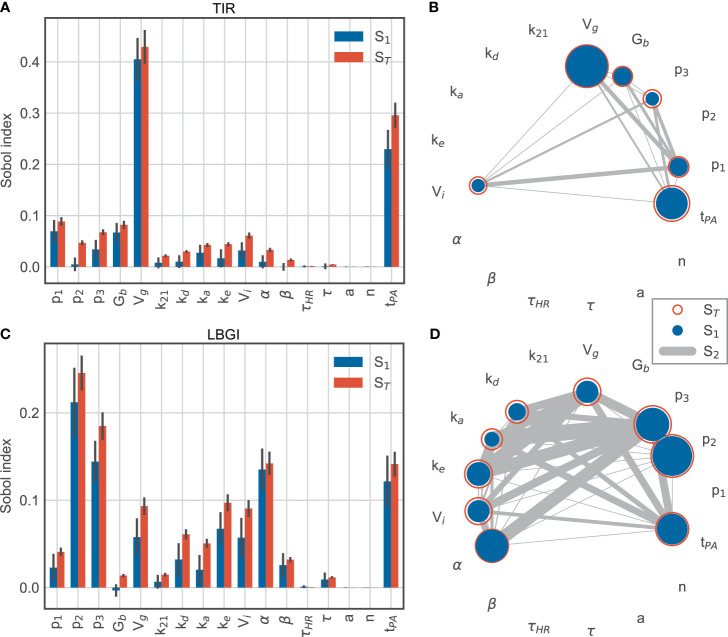
Sobol sensitivity indices for time-in-range (top row) and low blood glucose index (bottom row) for model parameters and exercise timing t_PA_. **(A, C)** main effect *S_1_* and total effect *S_T_*. **(B, D)** second-order effects S_2_ for parameters with a minimum total effect of 5%.

We extended our analysis by adding the timing of the exercise session as an additional parameter, where we allowed exercise to begin any time between 13:30h and 17:30h, and sampled *N* = 54,000 parameter sets. The exercise timing then accounts for more than 20% of the variation in TIR (**Figures 7A, B**) and its contribution is only exceeded by the glucose distribution volume *V_g_*. The relative contributions of the remaining parameters are very similar to before. In other words, the timing of exercise has a larger impact on time-in-range than all patient-related parameters, with the exception of *V_g_*.

For the LBGI, the relative contributions of the model parameters also remain similar to before, while the exercise timing now explains roughly the same amount of variation as α (**Figure 7C**). Overall, parameters involved in insulin action and exercise timing account for the majority of the variation in low blood glucose index.

These results confirm that exercise timing is an important contributor to the blood glucose dynamics, and that exercise-induced changes in insulin sensitivity appear as a major contributor to hypoglycemic events. The comparatively small contributions of interactions to the variation of LBGI show that exercise timing and insulin sensitivity provide independent contributions to this variation in the simulated scenario (**Figure 7D**).

## Discussion

In this study, we evaluated different proposed strategies for treatment adjustment to exercise in T1D *in-silico* using a mathematical model of glucose-insulin regulation and exercise metabolism. Simulation studies offer the opportunity to explore a broad range of possible scenarios and treatment options under identical conditions, and results can be systematically evaluated and compared. We relied on a combination of existing models describing glucose-insulin regulation during exercise, insulin kinetics and meal absorption, thus covering daily activities and allowing us to perform realistic long-term simulations.

We investigated the effect of exercise timing on BG dynamics, and tested a variety of combinations of exercise intensities and duration. For each scenario, we compared BG trajectories and corresponding measures of TIR, and LBGI and HBGI, which represent hypo- and hyperglycemia risk. Since exercise is associated with an increased risk of hypoglycemia during the activity, but also causes nocturnal hypoglycemic episodes due to a prolonged elevation of insulin sensitivity, we further studied acute and late-onset hypoglycemia risk for the different treatment adjustment strategies. Finally, we performed a global sensitivity analysis to determine the impact of model parameters, and hence individual processes such as insulin action, and exercise timing on TIR and LBGI.

We applied the CHO intake algorithm by Riddell and Milliken (29), which recommends the intake of fast-acting carbohydrates during exercise based on glucose readings and (downward) glucose trends. The aim of this algorithm is to keep glucose levels stable during the activity and avoid exercise-induced hypoglycemia. We studied glucose dynamics also after exercise when assuming no further adjustment of the remaining treatment. However, we are aware that the recommendations do not include treatment after exercise and do not target the prevention of late-onset hypoglycemia. Additionally, we used a set of consensus guidelines, which recommend treatment adjustment before, during and shortly after exercise (7). They provide recommendations on insulin bolus reduction for pre-exercise meals, starting glucose targets and CHO requirements during the activity, and we tested both the lower and upper carbohydrate recommendations.

The consensus guidelines (7) recognize the problem of exercise-related late-onset hypoglycemia, but cite only few related clinical studies. Due to this current lack of evidence, they do not provide differentiated guidelines for insulin dose adjustments and nutritional requirements based on intensity, duration, and timing of the activity for exercise of moderate duration and intensity. Similarly, late-onset hypoglycemia is discussed in the ISPAD guidelines (8), where it is further mentioned that no specific bedtime glucose guarantees the prevention of nocturnal hypoglycemia. We therefore did not apply any treatment adjustment strategy to reduce late-onset hypoglycemia but evaluated the BG outcome if detailed guidelines around the activity are followed. Treatment adjustments are nevertheless often made in clinical practice, but have to be based exclusively on experience rather than evidence-based guidelines.

Our simulation results suggest that the considered treatment adjustment strategies reduce acute hypoglycemia risk in general and can substantially reduce the increase in risk with exercise intensity and duration seen without adjustment. However, the risk for late-onset hypoglycemia remains elevated after exercise even with treatment adjustments for short and moderate exercise sessions, and can even exceed the risk after no adjustment if a correction bolus for post-exercise hyperglycemia is not reduced.

Our sensitivity analysis suggests that the prolonged rise in insulin sensitivity is the driving factor of late-onset hypoglycemia. Consequently, the increased insulin sensitivity should be considered for improving BG levels after exercise. Indeed, insulin and CHO requirements are usually adjusted for the rest of the day in clinical practice, and more targeted guidelines would be beneficial.

We could not observe clear trends regarding the risk of hyperglycemia, indicating that guidelines focus on prevention of hypoglycemia while accepting more hyperglycemic events to achieve this goal.

In our simulations, we observed that BG levels differ substantially during and after the same activity depending on timing of the exercise session in relation to meals. We hypothesize that the superimposed blood glucose dynamics of pre-exercise meal absorption, meal bolus and exercise make it difficult to derive a suitable treatment adjustment, and that the ongoing dynamic effect of exercise can lead to inadequate insulin bolus administration for post-exercise meals, where the nonlinear effect of increased insulin sensitivity affects larger insulin doses more. Consequently, late-onset hypoglycemia is more likely to occur after adjustment, with high CHO intake and thus elevated BG levels, compared to no adjustment for some scenarios. Overall, these findings suggest that it is difficult to avoid acute and late-onset hypoglycemia simultaneously if treatment after exercise is not adjusted appropriately and that the risk for late-onset hypoglycemia increases with more complex situations.

Recently, new guidelines on glucose management for exercise have been presented (34). They combine detailed guidelines on insulin treatment adjustment and CHO requirements before and during exercise and the immediate post-exercise period with information from CGM data, and take into account dropping and rising BG trends. Most likely, a tighter control of BG levels is achieved following this strategy. For the nocturnal period after late-afternoon or evening exercise, they propose intake of carbohydrates when glucose levels drop below a certain threshold. To target late-onset hypoglycemia proactively, this strategy could be combined with insulin sensitivity tracking. It was shown that insulin sensitivity can be estimated from CGM data (20, 21), and insulin doses could be scaled according to the exercise-driven change in insulin sensitivity compared to rest.

We first considered a single ‘typical’ person with diabetes for our analyses to allow direct comparison of different simulation scenarios and treatment guidelines. We then considered a random selection of subjects varying substantially in critical parameters and found that all results generalize to this setting.

We emphasize that it is not our aim to recommend actions for individuals, but to improve understanding of the effects of different treatment adjustments and their advantages and disadvantages. Our findings agree qualitatively with clinical observations on exercise-driven hypoglycemia (29, 35, 36) but are still predicated on the assumption that the model captures exercise processes adequately. In particular, we did not validate the full model in this study and our simulation scenarios extend substantially beyond the range of demonstrated validity for its individual model components. Our conclusions are therefore tentative in this respect, and further validation is warranted before application in a clinical setting.

Overall, we applied *in-silico* simulation studies as a useful tool for the systematic analysis and comparison of treatment strategies. We found that acute hypoglycemia can be prevented in most cases following current guidelines for treatment adjustment to exercise. Late-onset hypoglycemia presents an open problem and is caused by an elevated insulin sensitivity, where the timing of exercise in relation to meals plays a crucial role. Insulin bolus reduction of post-exercise meals might also be required depending on the timing of the exercise session. Similar studies could benefit the development of new treatment adjustments and the generation of testable clinical hypotheses, and validated models capturing additional physiological effects such as high intensity exercise and glycogen depletion during prolonged exercise would allow a broader range of exercise scenarios and strengthen conclusions based on in-silico simulations.

## Data Availability Statement

The Python code for model implementation, analysis, and reproduction of all figures can be found in the T1D Exercise Adjustment GIT repository at https://gitlab.com/csb.ethz/t1d-exercise-adjustment.

## Author Contributions

JD developed the Python code implementing the mathematical model, conducted simulations, and created the figures. H-MK and JD conceived and implemented the study and wrote the initial draft of the manuscript. SB, M-AB, and GS contributed in defining the simulation scenarios and provided clinical expertise in interpreting results. All authors contributed to the article and approved the submitted version.

## Funding

This work was supported by the two Cantons of Basel through project grant PMB-01-17 granted by the ETH Zurich.

## Conflict of Interest

The authors declare that the research was conducted in the absence of any commercial or financial relationships that could be construed as a potential conflict of interest.

## Publisher’s Note

All claims expressed in this article are solely those of the authors and do not necessarily represent those of their affiliated organizations, or those of the publisher, the editors and the reviewers. Any product that may be evaluated in this article, or claim that may be made by its manufacturer, is not guaranteed or endorsed by the publisher.
