# Supplementary material for: Simulation-Based Evaluation of Treatment Adjustment to Exercise in Type 1 Diabetes
Source: Front Endocrinol (Lausanne). 2021 Aug 19;12:723812. doi: 10.3389/fendo.2021.723812 (PMC8417413; doi:10.3389/fendo.2021.723812)
Supplement: Supplementary file 1 [file DataSheet_1.pdf]

Supplementary Material

1 SUPPLEMENTARY FIGURES

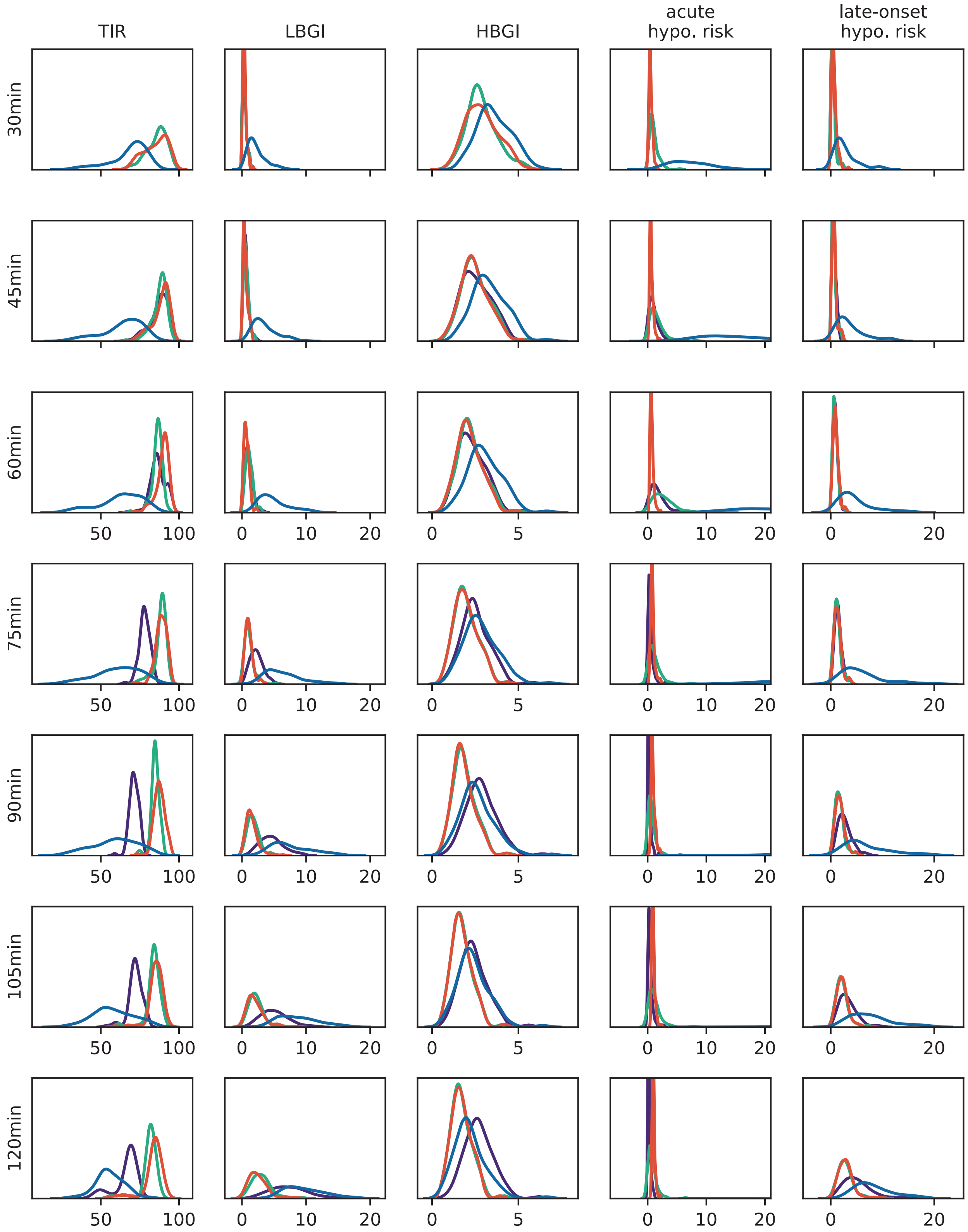

Figure S1. Distribution of TIR, LBGI and HBGI and the corresponding acute and late-onset hypoglycemia risk from 24h-simulations of a patient population. Exercise is performed with $HR = 140\,\mathrm{bpm}$ for different durations in the postabsorptive state (Scenario 1). No adjustment (blue), the CHO intake algorithm (orange), low (green) and high (purple) CHO recommendations are considered.

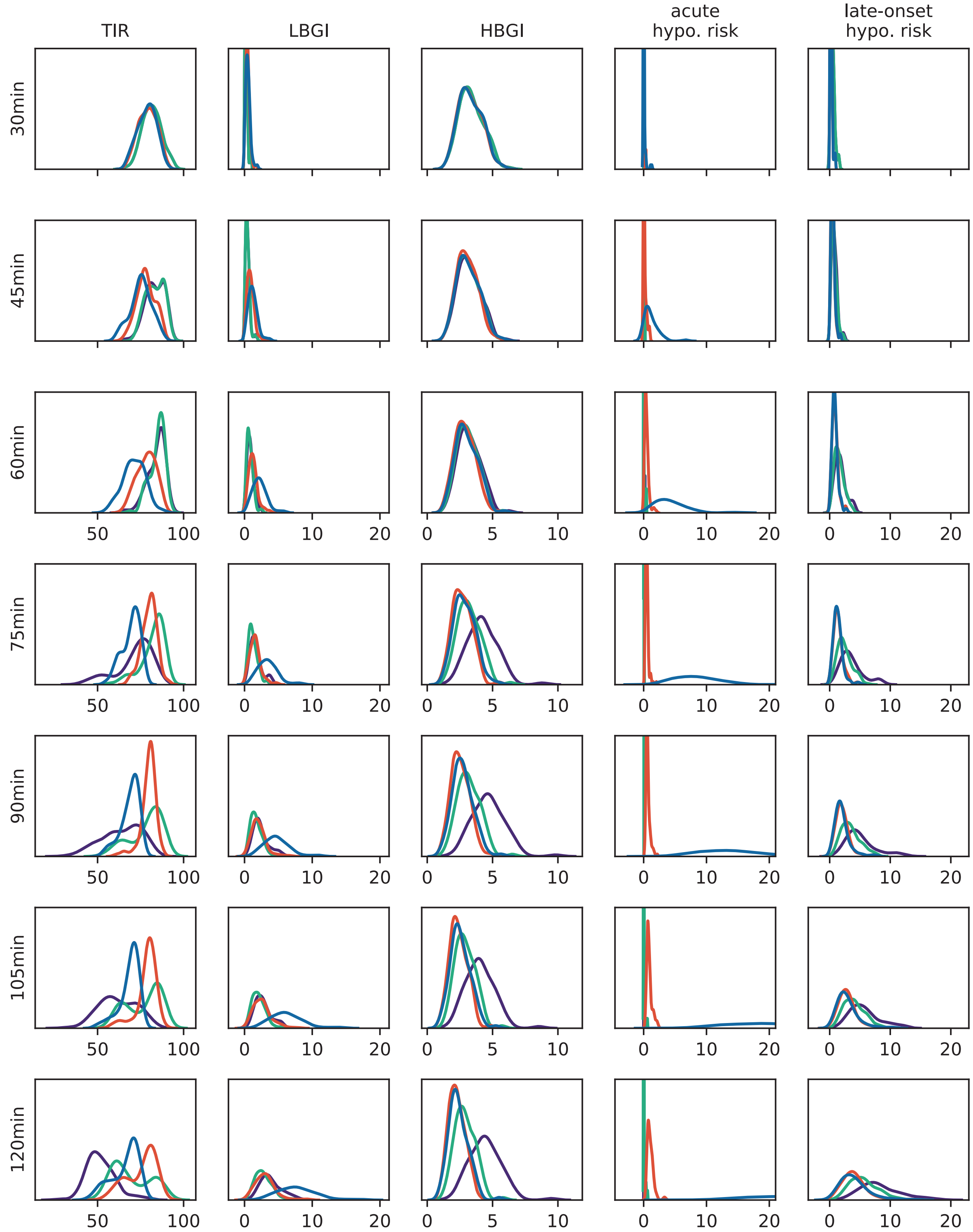

Figure S2. Distribution of TIR, LBGI and HBGI and the corresponding acute and late-onset hypoglycemia risk from 24h-simulations of a patient population. Exercise is performed with $HR = 140\,\mathrm{bpm}$ for different durations after a meal with insulin bolus reduction (Scenario 2). No adjustment (blue), the CHO intake algorithm (orange), low (green) and high (purple) CHO recommendations are considered.

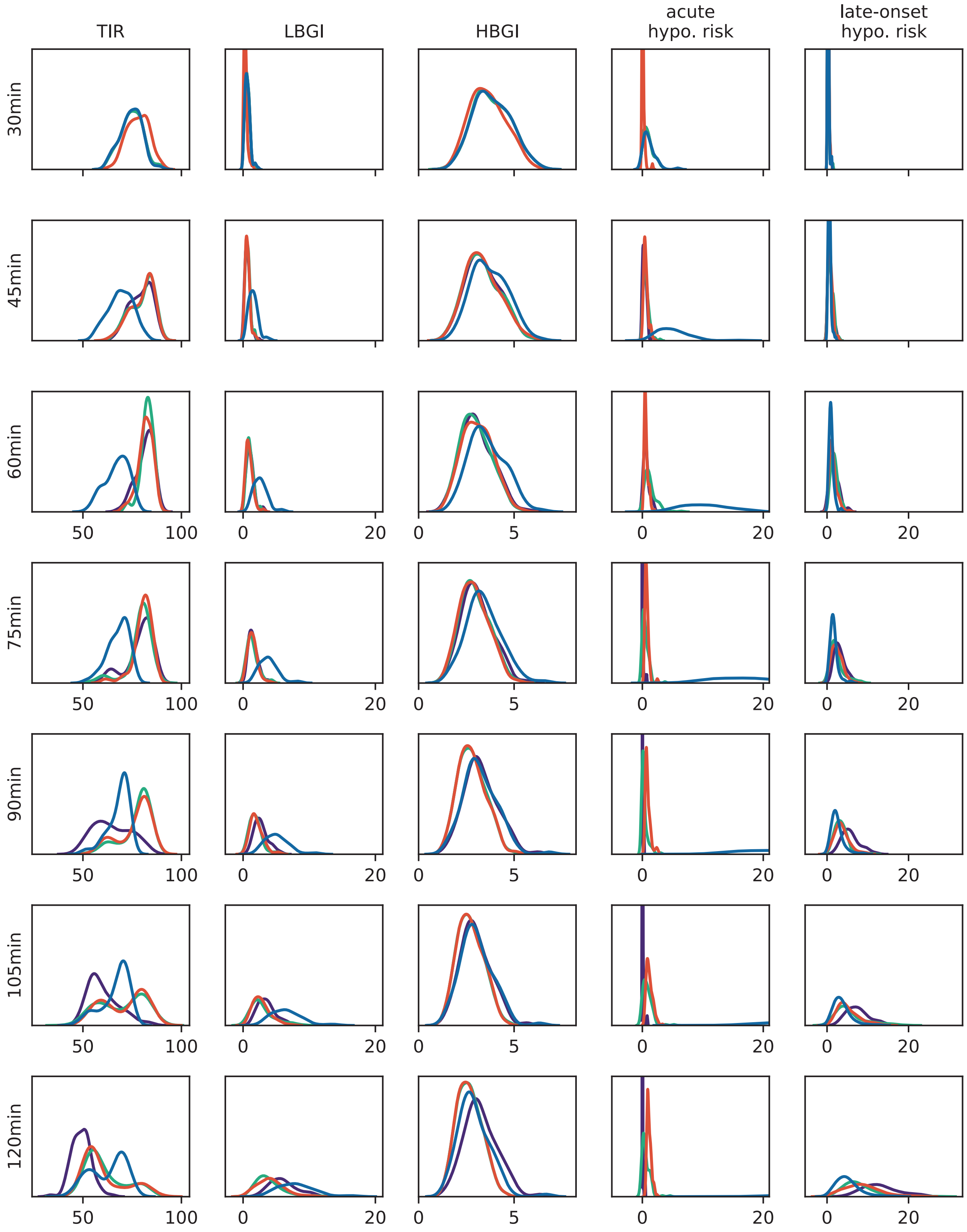

Figure S3. Distribution of TIR, LBGI and HBGI and the corresponding acute and late-onset hypoglycemia risk from 24h-simulations of a patient population. Exercise is performed with $HR = 140\,\mathrm{bpm}$ for different durations after a meal without insulin bolus reduction (Scenario 3). No adjustment (blue), the CHO intake algorithm (orange), low (green) and high (purple) CHO recommendations are considered.

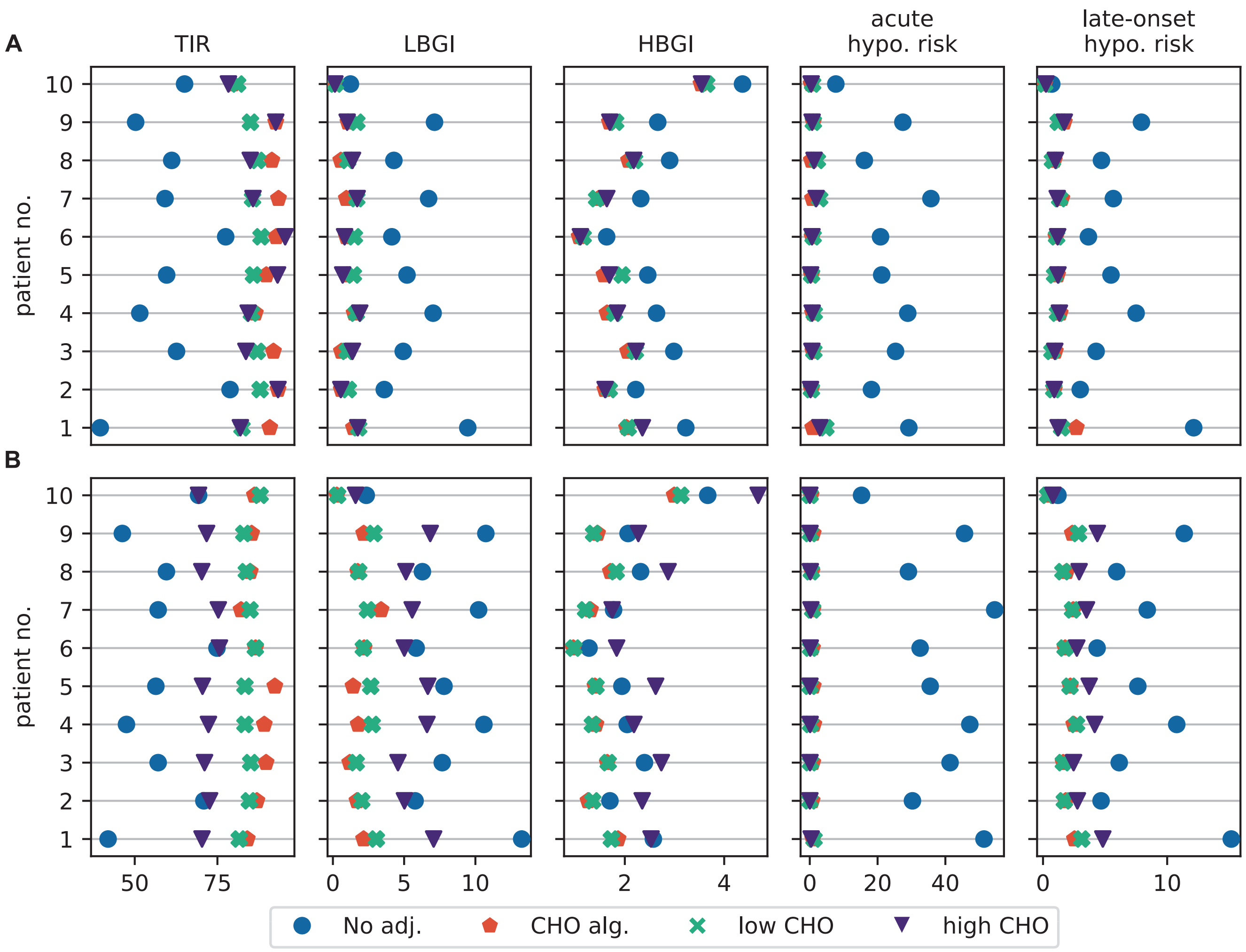

Figure S4. TIR, LBGI and HBGI over 24h-simulations and the corresponding acute and late-onset hypoglycemia risk for 10 subjects. Exercise is performed in the postabsorptive state (Scenario 1) for **(A)** 60 and **(B)** 90 min with $HR = 140\,\mathrm{bpm}$.

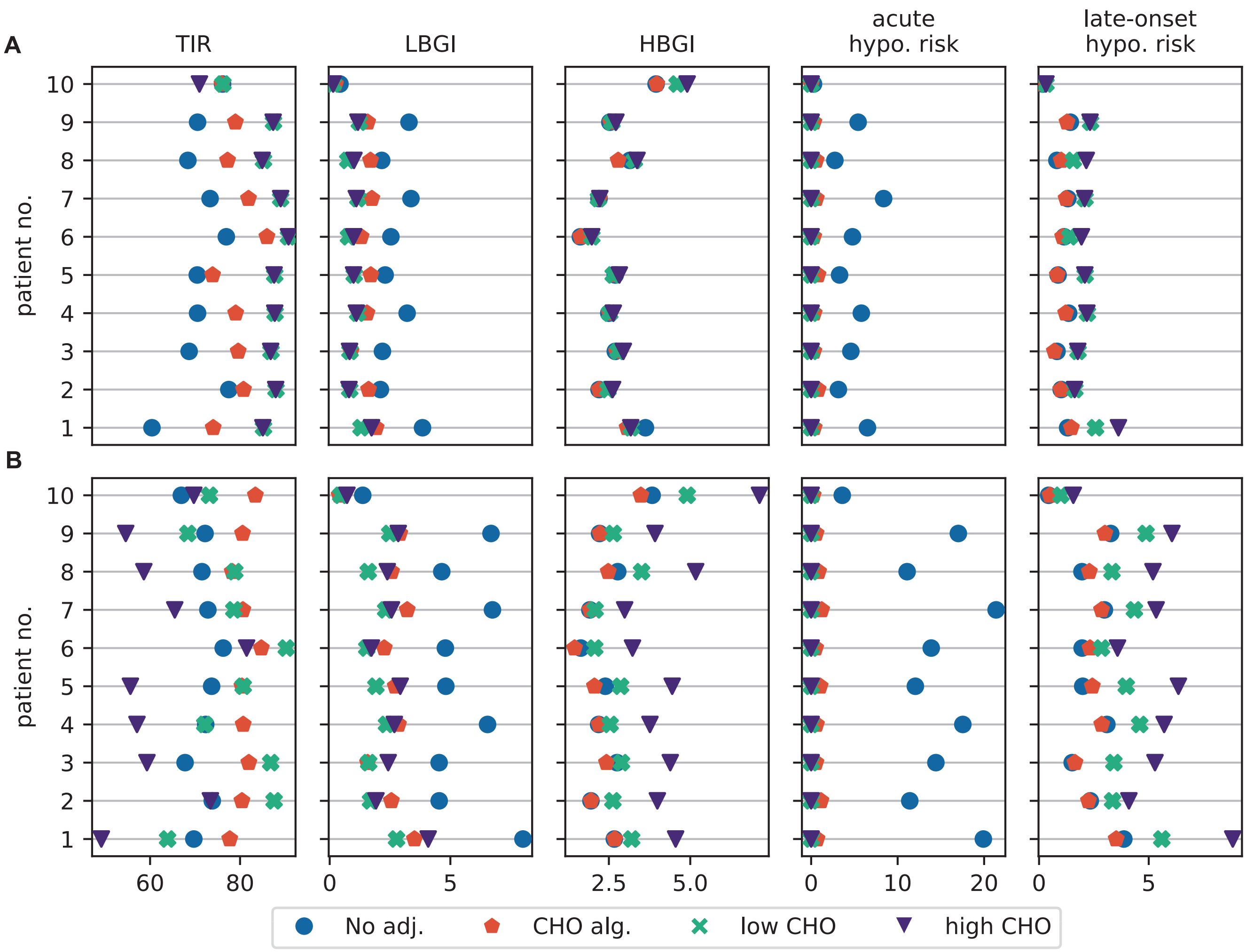

Figure S5. TIR, LBGI and HBGI over 24h-simulations and the corresponding acute and late-onset hypoglycemia risk for 10 subjects. Exercise is performed after a meal with insulin bolus reduction (Scenario 2) for **(A)** 60 and **(B)** 90 min with $HR = 140\,\mathrm{bpm}$.

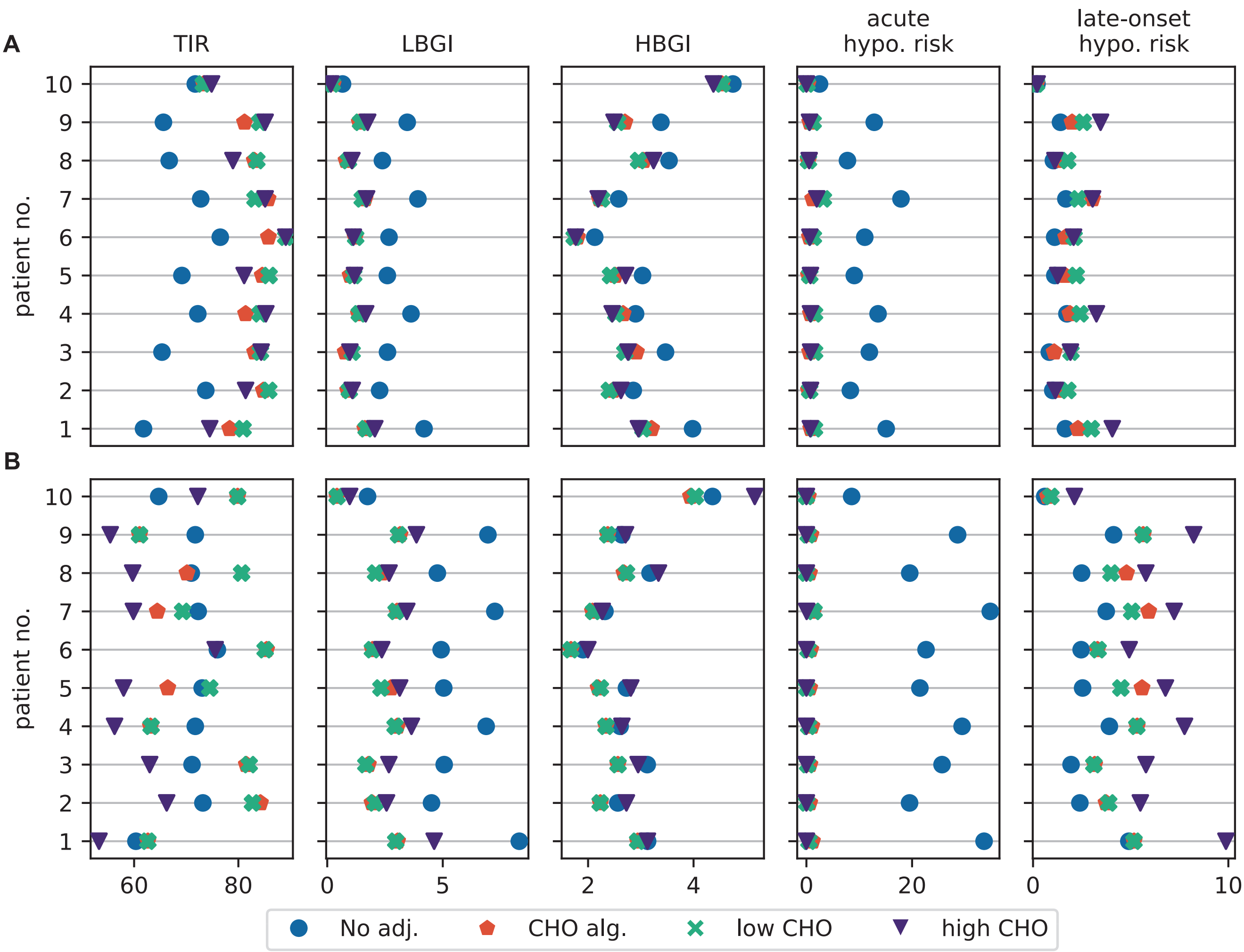

Figure S6. TIR, LBGI and HBGI over 24h-simulations and the corresponding acute and late-onset hypoglycemia risk for 10 subjects. Exercise is performed after a meal without insulin bolus reduction (Scenario 3) for **(A)** 60 and **(B)** 90 min with $HR = 140\,\mathrm{bpm}$.
